# Synthesis and Electrochemical Characterization of Nitrate-Doped Polypyrrole/Ag Nanowire Nanorods as Supercapacitors

**DOI:** 10.3390/ma17091962

**Published:** 2024-04-24

**Authors:** Hyo-Kyung Kang, Ki-Hyun Pyo, Yoon-Hee Jang, Youn-Soo Kim, Jin-Yeol Kim

**Affiliations:** 1 School of Advanced Materials Engineering, Kookmin University, Seoul 02707, Republic of Korea; rkdgyrud7274@kookmin.ac.kr (H.-K.K.); tnet98@kookmin.ac.kr (K.-H.P.); bada7371@kookmin.ac.kr (Y.-S.K.); 2 Advanced Photovoltaics Research Center, Korea Institute of Science and Technology (KIST), Seoul 02792, Republic of Korea; yhjang@kist.re.kr

**Keywords:** supercapacitor, silver nanowire–polypyrrole composite, nanorods, core–shell structure

## Abstract

Polypyrrole (PPy)-capped silver nanowire (Ag NW) nanomaterials (core–shell rod-shaped Ag NW@PPy) were synthesized using a one-port suspension polymerization technique. The thickness of the PPy layer on the 50 nm thickness/15 μm length Ag NW was effectively controlled to 10, 40, 50, and 60 nm. Thin films cast from one-dimensional conductive Ag NW@PPy formed a three-dimensional (3D) conductive porous network structure and provided excellent electrochemical performance. The 3D Ag NW@PPy network can significantly reduce the internal resistance of the electrode and maintain structural stability. As a result, a high specific capacitance of 625 F/g at a scan rate of 1 mV/s was obtained from the 3D porous Ag NW@PPy composite film. The cycling performance over a long period exceeding 10,000 cycles was also evaluated. We expect that our core–shell-structured Ag NW@PPy composites and their 3D porous structure network films can be applied as electrochemical materials for the design and manufacturing of supercapacitors and other energy storage devices.

## 1. Introduction

To develop efficient energy storage systems such as supercapacitors and batteries, the design of electrode materials and the manufacturing of active materials emerge as essential requirements [1,2,3,4,5,6]. Materials with high power density, long lifecycle, and fast charge–discharge characteristics are key requirements for these energy storage devices. In particular, redox-active materials [7,8] comprising π-conjugated bonded polymers or inorganic oxides (such as ruthenium oxide) [9] have been mainly used as active materials in supercapacitors. In the case of inorganic oxides, the charge storage mechanism involves only the surface of the oxide particles because their capacity is limited by surface area, while redox-active polymers have the advantage of being designed to ensure that the entire volume of the polymer is involved in the charge storage process, and their capacities are much higher than those of oxide devices. Other advantages of redox-active polymers include low cost, high conductivity, chemical stability, and ease of designing nanostructures with large surface areas. However, the charge storage capacity and energy density of energy storage systems, especially supercapacitors, still require further improvement. Recently, polyaniline and polypyrrole (PPy), which are π-conjugated polymers, have been widely studied because they have excellent conductivity, are very stable in air, and are advantageous for manufacturing film-type cells or ultralight cells [7,8,9,10]. In particular, conductive carbon particles, carbon nanotubes, and graphene, which form a conductive network from a complex combined with a conductive polymer, and materials with electrochemically improved physical properties have emerged as subjects of greater interest [11,12,13,14,15,16]. In addition, effective properties can be utilized using nanosized metals or metal oxides, such as iron, manganese, cobalt, or vanadium oxide, and iron oxide as hybridization materials with π-conjugated polymers, which enhance ion diffusion throughout the material and ultimately oxidation [4,17].

Recently, carbon-based nanomaterials or their hybrid structures with metals have been attracting attention as electrode active materials for supercapacitor applications. These nanomaterials are reported to have a very high surface area, control over the porous structure, excellent electrical conductivity, chemical inertness, and excellent electrochemical properties [18,19,20]. However, although carbon-based materials have been very successful in supercapacitor applications, the achieved energy densities are still low, below 10 Wh/kg. In particular, in the case of conductive polymers, higher energy densities can be achieved, but they are known to be vulnerable to long-term stability [21]. In particular, in this study, because the one-dimensional PPy-Ag NW hybrid nanomaterial is networked and based on a porous structure with a 3D film structure, it can have the following superior structural characteristics [21]: (a) an optimized average pore size (micro–meso pores) for superior charge storage and efficient transport of electrolyte ions for improving energy density and maintaining superior power densities, respectively, (b) a large number of adsorption and active sites which can be expanded for pseudo-capacitive charge storage, (c) improved surface chemistry to enhance electrolyte wettability for good power capabilities, and (d) a stable and highly conductive platform for long-term stabilities.

In this study, we successfully synthesized nitrate-doped PPy-capped silver nanowire (Ag NW) nanorod-shaped architectures (Ag NW@PPy) using a simple suspension polymerization method and observed high-performance supercapacitor properties with cycling stability. The Ag NWs surrounded by nitrate-doped PPy formed one-dimensional (1D) structures with the structural characteristics of a core–shell structure. More importantly, the Ag NWs were first hybridized with a pseudomaterial (PPy) and then assembled into electrodes, and the two materials were tightly bonded in a core–shell structure. The core–shell structure was characterized by a coaxial conductive PPy layer as the outer shell and a highly conductive Ag NW core on the inside. PPy was chosen because it has high electrical conductivity and can enable rapid redox reactions and stable storage/charge processes, and Ag NWs with excellent conductivity were used to improve the capacitance [22,23,24]. However, the incorporation of Ag NWs into the PPy-capped structure resulted in improved electrical conductivity, and the Ag NWs acted as efficient conductive pathways, facilitating rapid charge transfer and reducing the internal resistance. The core–shell design of Ag NWs wrapped by PPy provides a high aspect ratio, and this elongated geometry enhances the surface area available for charge storage, leading to increased capacitance. Here, the Ag NW@PPy nanocomposite is a 1D core–shell-structured heterojunction material that is a hybrid of Ag NW with excellent electrical conductivity and conductive PPy. Well-defined heterojunction interfaces can enhance charge transfer, improve electron mobility, and promote redox reactions. Chen et al. [25] reported that metal additives such as plasmonic Ag can exhibit high electrochemical performance in heterojunction nanostructures.

In summary, supercapacitors using these core–shell Ag NW@PPy nanostructures can achieve remarkable performance in terms of electrical conductivity and capacitance. We obtained high specific capacitances of 625 F/g at a scan rate of 1 mV/s from the Ag NW@10 nm PPy samples and evaluated the cycling performance of 3D porous Ag NW@PPy composite films over 10,000 cycles. We also expect that these 3D porous Ag NW@PPy structure network films can be applied as electrochemical materials for the design and manufacturing of supercapacitors and other energy storage devices.

## 2. Materials and Methods

### 2.1. Synthesis of Core–Shell Ag NW@PPy Nanorods

**Scheme 1:**
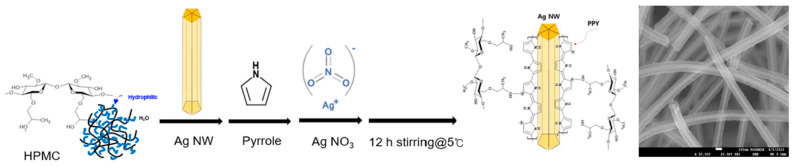
Illustrative representation of the synthesis process of the core–shell-structured Ag NW@PPy nanorods and their scanning electron microscopy (SEM) image.

Pyrrole monomer (Py; ≥99%), silver nitrate (AgNO_3_; 99.8%), hydroxypropyl methyl cellulose (HPMC; 99.8%), polyvinylidene fluoride (PVDF), and *N*-methyl-2-pyrrolidone (NMP; 98%) were purchased from Sigma-Aldrich (St. Louis, MI, USA). All reagents were used without further purification. The Ag NWs were synthesized according to the polyol method, as described in a previous paper [26]. Synthesis of the core–shell rod-shaped Ag NW@PPy nanohybrid materials was performed using a simple one-port suspension polymerization technique, as shown in Scheme 1. Briefly, Ag NWs (0.5 wt%, 1 mL aqueous solution) were first dispersed in a mixed water/ethanol solution (1:5 ratio *v*/*v*, 30 mL) with 1 wt% HPMC dispersed in it. Then, 0.25 g of pyrrole monomer was added and stirred at 0 °C for 30 min. Finally, 0.63 g of AgNO_3_, which is both a dopant and an oxidizing agent, was added to the mixture and reacted at 0 °C for 12 h to complete the synthesis of the final solution. The reaction products were purified using a 0.2 µm PTFE filter, washed thrice with deionized water and ethanol, and dried in an oven at 100 °C.

### 2.2. Electrochemical Performance Evaluation and Measurement

First, the purified Ag NW@PPy powder was mixed with activated carbon (super P), SWCNT, and PVDF binder in a ratio of 7:1.5:0.5:1 under NMP solvent conditions, and a slurry was formed using a ball mill (450 rpm, 2 h). The resulting slurry was uniformly coated onto an Al foil substrate (test cell with three electrodes) with a thickness of approximately 15 µm and dried in a vacuum oven at 60 °C for more than 12 h to produce an electrode plate. Then, the working electrodes were constructed by pressing the Ag NW@PPy/super P/SWCNT/PVDF composite onto an Al foil surface with an area of 0.3 cm^2^. To fabricate the cell, a porous polypropylene membrane was used as the separator, and a liquid electrolyte containing LiPF_6_ dissolved in an ethylene carbonate complex solvent at a concentration of 1.15 M was used as the electrolyte. Finally, electrochemical properties, including cyclic voltammetry (CV) curves and galvanostatic charge/discharge (GCD) characteristics, were measured using a potentiostat/galvanostat autolab electrochemical workstation in a three-electrode configuration. All experiments were performed at room temperature. Pt wires and Ag/AgCl (3 M, KOH) were used as the counter electrodes and reference electrode, respectively. CV and GCD measurements were performed using a multichannel potentiostat/galvanostat system. The specific capacitance of the fabricated electrode was calculated from GCD curves. The morphology, microstructure, and electronic absorption of the core–shell Ag NW@PPy nanorods were investigated via scanning electron microscopy (SEM; JSM-633F, Jeol), Fourier-transform (FT) Raman spectroscopy (Renishaw InVia Microscope, UK), and standard four-probe measurement (Loresta, Mitsubishi Chemical), respectively.

## 3. Results and Discussion

**Figure 1:**
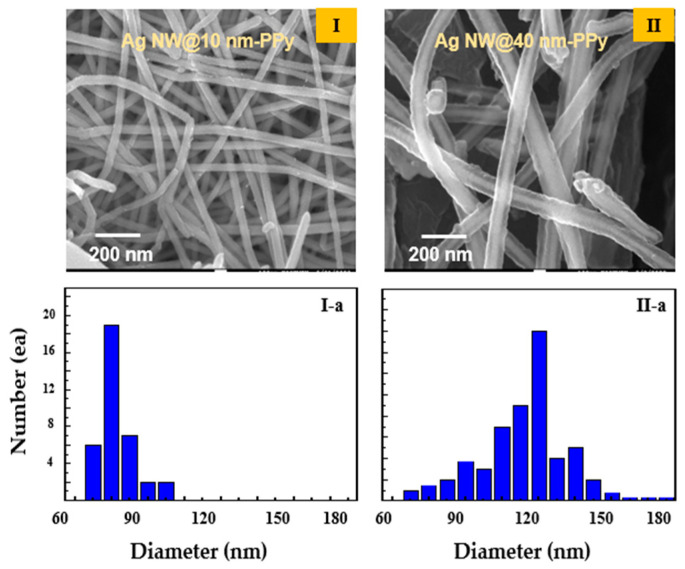
SEM morphologies of the core–shell rod-shaped nanocomposites (Ag NW@PPy). (**I**) Ag NW@PPy nanocomposite surrounded by a PPy layer of approximately 10 nm (sample 1) on the outer surface of the Ag NWs with a diameter of 50 nm and a length of 15 µm and (**I-a**) its diameter distribution. (**II**) Ag NW@PPy nanocomposite surrounded by a PPy layer of approximately 40 nm (sample 2) and (**II-a**) its diameter distribution.

Core–shell rod-shaped Ag NW@PPy nanomaterials were successfully synthesized using a simple suspension polymerization technique, as shown in Scheme 1. In this study, Ag NWs were dispersed in an aqueous solution (0.1 wt%) and mixed with pyrrole monomer to create pseudomaterials, and Ag NW@PPy was prepared via suspension polymerization. As a result, a 1D Ag NW@PPy with a core–shell structure was created, in which a thin layer composed of conductive PPy chains was assembled as an active material on the surface of the Ag NW, as shown in Figure 1. Structurally, during polyol synthesis, the 1D Ag NWs were thinly capped with a polymeric surfactant containing CO groups on the surface so that they could chemically bond with the NH groups present in the conductive PPy chains [26]. The thickness of the PPy layer was controlled by the amount of pyrrole monomer added during suspension polymerization (Scheme 1). Here, PPy refers to conductive PPy doped with nitrate, and it was grown as a thin layer through the polymerization process of pyrrole monomer on the outer surface of 50 nm thick Ag NW. At this time, PPy was interconnected to the Ag surface through charge–charge interaction. In fact, it was observed that the critical order and electrical conductivity of PPy hybridized with Ag NW were improved, and the band gap energy resulting from the π-π* transition of the conjugated PPy chain was also reduced. In fact, as PPy was chemically linked with Ag NW, it was observed that the relative intensity of the C–C/C=C and C–H/C–N stretching modes due to the pyrrole ring of PPy increased with the combination of Ag NW. Specifically, PPy, a π-conjugated polymer, is the redox material and is used as the main active material in supercapacitors. Following the procedure in Scheme 1, 1D Ag NW with a thickness of 50 nm and a length of 15 µm were used, and the PPy layers polymerized to them were 10 nm (sample 1, Ag NW@10 nm PPy), 40 nm (sample 2, Ag NW@40 nm PPy), 50 nm (sample 3, Ag NW@50 nm PPy), and 60 nm (sample 4, Ag NW@60 nm PPy). In rod-shaped Ag NW@PPy, PPy has been shown to significantly improve the electrical conductivity by more easily transferring electrons to the metallic Ag NW and expanding the electron transport path. However, a 1D material consisting of a highly conductive Ag NW core and a high-capacity PPy shell can exhibit properties that can improve the electrochemical performance of supercapacitor devices by improving the charge transport ability and reducing the overall electrical resistance.

Figure 1(I,II) show the SEM images and diameter distributions of two samples of the synthesized Ag NW@PPy nanocomposites. The SEM images of the Ag NW@PPy nanocomposites show a well-defined core–shell structure, with the Ag NWs impregnated into PPy in the form of single coaxial nanorods. Their structures exhibited 1D morphologies with a length of approximately 15 µm and average diameters of 70 nm (Figure 1(I), sample 1) and 130 nm (Figure 1(II), sample 2). The Ag NW@PPy nanocomposites were surrounded by PPy layers of approximately 10 nm (sample 1) and 40 nm (sample 2) on the outer surface of the Ag NWs, with a diameter of 50 nm and a length of 15 µm.

**Figure 2:**
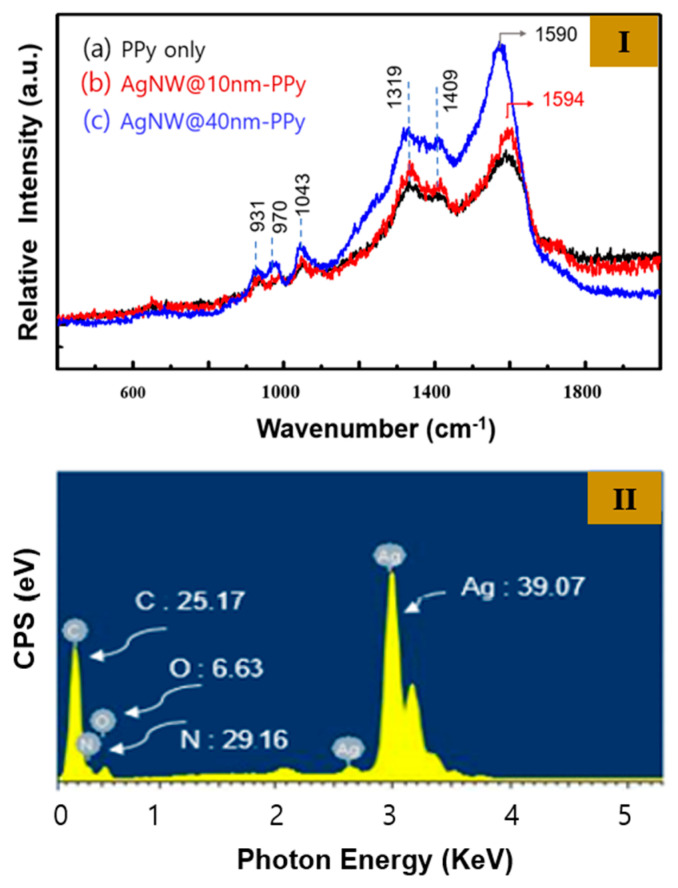
(**I**) FT Raman spectra of the Ag NW@PPy nanorods (samples 1 and 2): (a) pure PPy particles, (b) Ag NWs with PPy capped to a thickness of 10 nm (Ag NW@10 nm PPy), and (c) Ag NWs with PPy capped to a thickness of 40 nm (Ag NW@40 nm PPy). (**II**) EDX spectra of sample 2.

Figure 2(I) shows the Raman data measured for the structural analysis of sample 1 and 2. Through Raman spectroscopic analysis, we were able to clearly prove the molecular structure of PPy doped with nitrate (NO_3_^−^) surrounding the outer side of the Ag NWs. The Ag NW@PPy nanorods were prepared by suspension polymerization on the Ag NW surface through a facile redox reaction between the pyrrole monomer and silver nitrate. During the redox reaction, the pyrrole monomer and silver nitrate acted as the reducing agent and dopant, respectively. The pyrrole monomer bound to the Ag NW surface polymerized with the dopant (nitrate) to form a thin layer on the Ag NW, and trace silver particles were generated and adsorbed on the PPy polymer surface. As a result, a Ag NW@PPy with a core–shell structure surrounded by nitrate-doped PPy on the Ag NW surface was formed. Figure 2(I-a) shows the Raman data obtained from pure PPy nanoparticles, and Figure 2(I-b,I-c) show the data obtained from samples 1 and 2, respectively, which were surrounded by PPy with thicknesses of 10 and 40 nm (Ag NW@10 nm PPy and Ag NW@40 nm PPy), respectively. In the Raman spectrum of the obtained Ag NW@PPy, the peaks at 1590–1594, 1409, 1319, 1043, 970, and 931 cm^−1^ are attributed to the C–C/C=C and C–H/C–N stretching bands due to the quinoid structure of the pyrrole ring, the C–N stretching of the pyrrole ring, the stretching of the positively charged C–NH^+^ group, and the C–H out-of-plane pyrrole ring. In particular, the Raman spectrum of PPy is dominated by a band at approximately 1319 cm^−1^, which is assigned to the positively charged C–NH^+^ stretching mode of the pyrrole ring generated by doping. In the figure, the C–C/C=C and C–H/C–N stretching modes due to the pyrrole ring in the range of 1319–1594 cm^−1^ show an increasing trend in their relative intensity in the Ag NW@PPy. In addition, their relative intensity increases with blue shift, proportional to the PPy thickness.

Figure 2(II) shows the energy dispersive X-ray spectroscopy (EDX) spectra of sample 2. These are the EDX data measured to analyze the composition of each atom constituting the Ag NW@PPy nanorods. The mass percentage of the Ag atoms was specifically estimated and compared with the contents of other components. In addition, the ratio of nitrogen to oxygen atoms newly created by doping was estimated. Based on the analysis, the weight contents of the major atoms such as C, O, N, and Ag were 25.17%, 6.63%, 29.16%, and 39.07%, respectively. Particularly notable was the 6.63% of oxygen atoms, as they do not exist in PPy molecules, and nitrogen atoms were confirmed to be present in excess. These were interpreted as originating from the dopant (nitrate).

**Figure 3:**
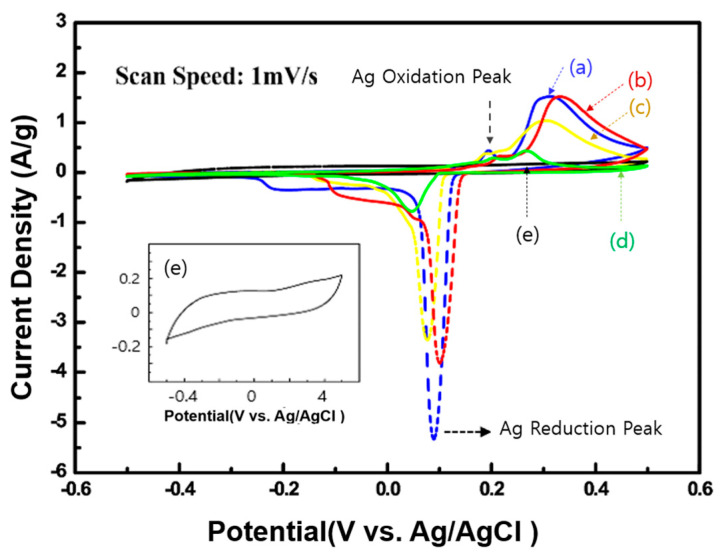
CV curves of the Ag NW@PPy nanorods with a molecular weight of 2 mg/electrode at a scan rate of 1 mV/s in 1 m KOH: (a) Ag NW@10 nm PPy (blue), (b) Ag NW@40 nm PPy (red), (c) Ag NW@50 nm PPy (yellow), (d) Ag NW@60 nm PPy (green), and (e) pure PPy (black, as a reference).

**Figure 4:**
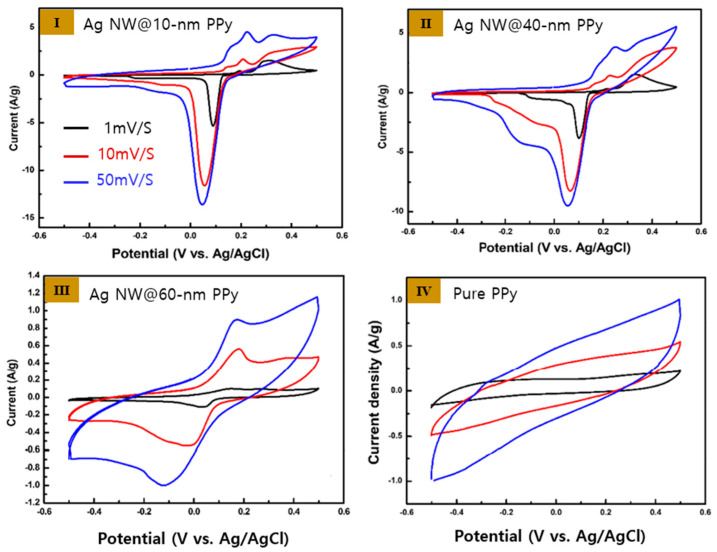
CV curves of the Ag NW@PPy nanorods obtained at scan rates of 1 mV/s (black), 10 mV/s (red), and 50 mV/s (blue): (**I**) Ag NW@10 nm PPy, (**II**) Ag NW@40 nm PPy, (**III**) Ag NW@60 nm PPy, and (**IV**) pure PPy.

**Figure 5:**
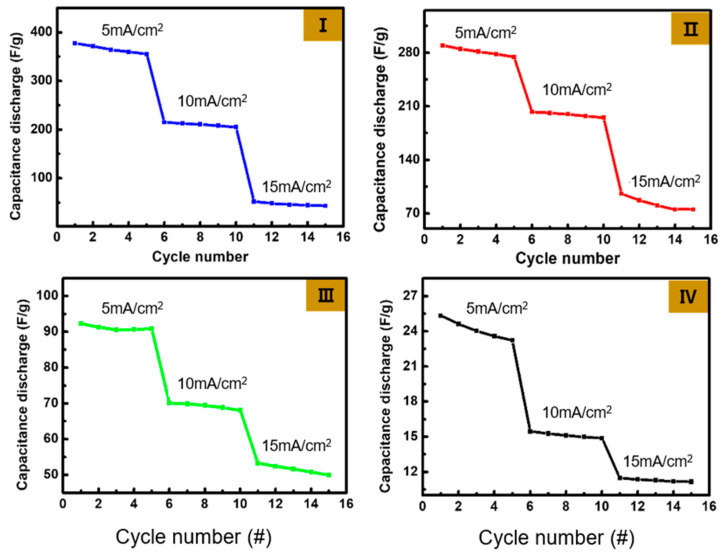
GCD characteristics of the Ag NW@PPy nanorod supercapacitor cells measured at scan rates of 5, 10, and 15 mA/cm^2^: (**I**) Ag NW@10 nm PPy, (**II**) Ag NW@40 nm PPy, (**III**) Ag NW@60 nm PPy, and (**IV**) pure PPy.

The synthesized Ag NW@PPy powder was mixed with super P, SWCNT, and PVDF binder in an NMP solvent to prepare a slurry, and the composited slurry materials were coated on a Cu foil. Under the three-electrode system, Pt and Ag/AgCl were used as the counter and reference electrodes, respectively. CV curves were first observed in 1 m KOH at scan rates between 1 and 50 mV/s, as shown in Figure 3, Figure 4 and Figure 5. The Ag NW@PPy nanorod materials were evaluated under various conditions to confirm their applicability and charge/discharge characteristics as electrochemical supercapacitors. The experiments were particularly focused on evaluating the effect of the PPy layer thickness difference on the capacitance of the Ag NW@PPy nanorods. Figure 3 shows the CV properties of the four synthesized Ag NW@PPy nanorod samples (Ag NW@10 nm PPy, Ag NW@40 nm PPy, Ag NW@50 nm PPy, and Ag NW@60 nm PPy) and pure PPy (as a reference). Measurements were made in the range of –0.5 to +0.5 V at a scan rate of 1 mV/s, and the capacitance was observed according to changes in the thickness of the PPy layer. The CV curves derived from all samples showed not only distinct pseudo-capacitive behavior but also well-defined differences, which were attributed to oxidation and reductions. Two distinct peaks were measured at 0.20–0.32 V and 0.02–0.10 V, which were attributed to oxidation and reduction reactions, respectively [27,28]. As shown in Figure 3, when the Ag NW was complexed with PPy, their redox potential greatly improved by more than five times that of pure PPy, which was interpreted as the effect of Ag contained in Ag NW@PPy. This indicates that Ag NW@PPy has higher electrical conductivity than pure PPy, allowing for greater current flow. In addition, when 1D Ag NW@PPy was coated on Al foil, the film morphology formed a 3D porous structure with a large surface area. Moreover, the porous form of this 3D structure could provide a larger surface area for interaction with electrolyte ions in the cell. Therefore, it can be proven that Ag NW@PPy with a 1D structure will store more charges than pure PPy. The inset in Figure 3 shows an enlarged version of the CV curve of pure PPy. However, in Ag NW@PPy, when the PPy thickness was greater than 60 nm, there were few peaks due to the redox reaction of Ag.

Figure 4 shows the CV curves of the Ag NW@PPy nanorods obtained at scan rates of 1, 10, and 50 mV/s. As shown in the figure, as the scan rate increased, the area of the CV peak tended to increase significantly. In addition, the anodic (oxidation) peak tended to shift to higher voltages, whereas the cathodic (reduction) peak tended to shift to lower voltages. In all Ag NW@PPy samples, the redox response of Ag was confirmed even at high scan rates. In contrast, in pure PPy, it was confirmed that only the ion transfer reaction occurred on the surface. In addition, when the PPy thickness was increased to 60 nm, the flowing current density tended to decrease significantly, and the redox reaction of Ag decreased sharply. However, the main advantage of the Ag NW@PPy nanocomposites is that the charge transfer between the Ag NW in the core part and the PPy shell layer occurs efficiently. Electrolyte ions moved between the large openings of the 3D microporous structure of the nanocomposites, and these nanocomposites exhibited excellent electrochemical performance. The CV results of the Ag NW@PPy nanocomposite devices with different PPy shell thicknesses showed that as the PPy layer thickness increased, the current density flowing through the device decreased and the redox potential by Ag decreased significantly.

The charge storage properties of Ag NW@PPy were observed through GCD curves at various scan rates. The data measured at scan rates of 5, 10, and 15 mA/cm^2^ are shown in Figure 5. Figure 5 also shows the GCD characteristics and cycling test results of three Ag NW@PPy nanorod samples and a pure PPy reference sample. GCD measurements were performed in 1 m KOH electrolyte. Figure 5(I) shows the GCD characteristics obtained from the Ag NW@10 nm PPy sample. The charge and discharge curves are approximately symmetric and exhibit stable discharge/discharge capacitance. The discharge time was observed to be approximately 50 s at a 5 mA/cm^2^ scan rate, and stable charge/discharge characteristics were maintained for five cycles. On the contrary, at a scan rate of 15 mA/cm^2^, the discharge time was slower (to over 100 s). In addition, as the scan rate increased to 10 mA/cm^2^ and 15 mA/cm^2^, the discharge capacitance value decreased relatively significantly, as shown in Figure 5. According to the Butler–Volmer equation [23], the lower the current density, the lower the side reaction and the shorter the discharge time. Figure 5(II, III) show the GCD curves obtained from the Ag NW@40 nm PPy and Ag NW@60 nm PPy samples, respectively. As can be seen in the figure, Ag NW@40 nm PPy and Ag NW@60 nm PPy exhibit more reduced discharge capacity at all scan rates compared to Ag NW@10 nm PPy. However, the discharge capacitance of Ag NW@PPy tended to reduce as the thickness of the PPy layer increased, and the smallest result was obtained for Ag NW@60 nm PPy. In addition, in Ag NW@60 nm PPy, the influence of Ag oxidation/reduction was greatly reduced, and more stable GCD curves were obtained. Nevertheless, all values obtained from Ag NW@PPy were higher than those obtained from pure PPy. As a result of the cycling test, the most stable results were obtained at scan rate of 10 mA/cm^2^.

**Figure 6:**
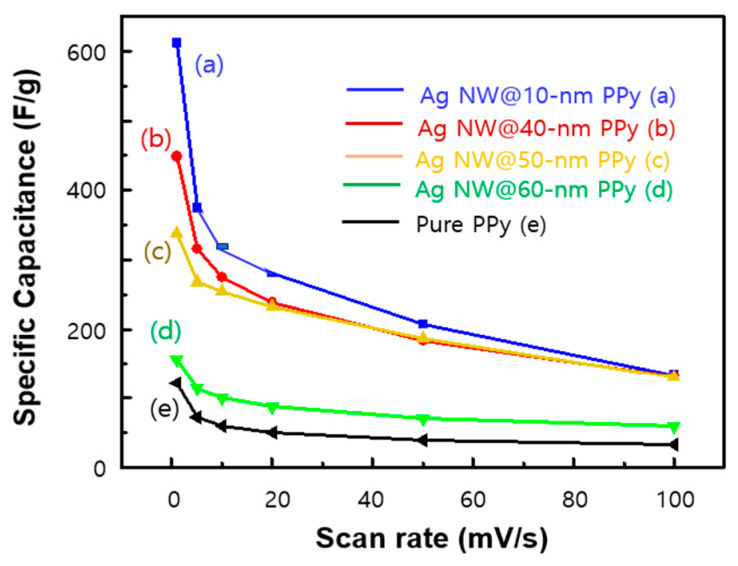
Change in specific capacitance (F/g) according to the scan rate: (a) Ag NW@10 nm PPy, (b) Ag NW@40 nm PPy, (c) Ag NW@50 nm PPy, (d) Ag NW@60 nm PPy, and (e) pure PPy sample.

On the basis of the GCD data analysis, the specific capacitance (F/g) according to the scan rate was calculated, as shown in Figure 6. As shown in Figure 6a, for the Ag NW@10 nm PPy sample, the specific capacitances were 625, 372, 325, and 152 F/g at scan rates of 1, 5, 50, and 100 mV/s, respectively. For the Ag NW@60 nm PPy sample (Figure 6d), the specific capacitances tended to decrease sharply to 165, 120, 80, and 75 F/g, respectively, which were higher than those of the pure PPy sample (Figure 6e). The specific capacitance decreased as the scan rate increased because the diffusion of electrolyte ions inside the PPy layer became more difficult when the scan rate was fast and, as a result, the capacitance of the cell decreased. In any case, the Ag NW@PPy cells showed improved specific capacitances compared to the pure PPy cell and, in particular, the specific capacitance appeared to be greatly affected by the thickness of the PPy layer. This indicates that the 3D porous Ag NW@PPy layer with a higher protonation level and better electrical conductivity is more suitable for the adsorption/desorption of electrolyte ions.

**Figure 7:**
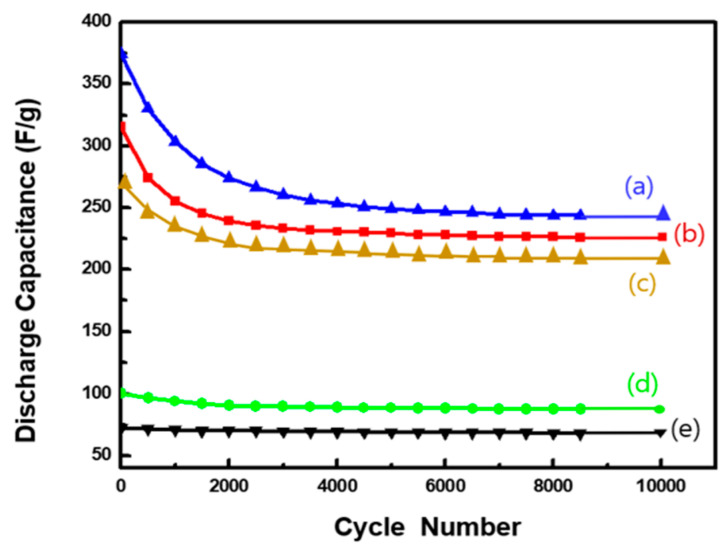
Cycling performance of the discharge capacitance measured at a scan rate of 5 mA/cm^2^ and 25 °C: (a) Ag NW@10 nm PPy, (b) Ag NW@40 nm PPy, (c) Ag NW@50 nm PPy, (d) Ag NW@60 nm PPy, and (e) pure PPy sample.

Figure 7 shows the results of the cycling performance evaluation of the discharge capacitance of the Ag NW@PPy composite samples measured at a scan rate of 5 mA/cm^2^. We evaluated the cycling performance of the 3D porous Ag NW@PPy composites over a long period of time (over 10,000 cycles) as an important key parameter for supercapacitor performance. The Ag NW@PPy composite samples with PPy thicknesses of 50 nm or less initially showed high irreversible discharge capacitance, but after 2000 cycles, the decrease in discharge capacitance tended to become smaller and stabilized. On the contrary, in the case of the Ag NW@60 nm PPy composite, a slight change in discharge capacitance and stabilized characteristics were observed overall, except for the initial slight irreversible discharge capacitance, as shown in Figure 7d. In any case, it has been proven that the discharge capacitances obtained from all of the Ag NW@PPy composite samples are higher than those of pure PPy (Figure 7e). In particular, although the reasons for the superior cycling stability of Ag NW@60 nm PPy have not yet been fully proven, we propose two important factors that may contribute to the enhancement of cycling stability when the thickness of the PPy layer is greater than 60 nm: (1) in the core–shell structure, a thicker surface layer composed of PPy can reduce the charge resistance and shorten the diffusion path of ions across the surface, and (2) the well-polymerized PPy nanostructures on the core–shell surface provide enhanced mechanical strength to the nanohybrid structure, which can maintain long-term reversible reactions while maintaining the stability of the electrode.

The Ag NW@PPy nanocomposite is a 1D core–shell-structured heterojunction material that is a hybrid of Ag NW with excellent electrical conductivity and conductive PPy. In particular, in the case of the Ag NW@PPy nanocomposite, the heterojunction interface between Ag NW and PPy is chemically linked by charge–charge interaction, which can improve charge separation and promote electron transfer, contributing to improved electrochemical performance. It acts as a bridge for efficient electron transfer between Ag NWs and PPy. Here, Ag NWs serve to enhance electrochemical activity by enhancing ion and electron transport. But, as shown in the figure, when the PPy is capped thinly with a thickness of 50 nm or less, the discharge capacity is reduced at the beginning of the cycle test. As a result of XRD analysis, it was confirmed that this was because part of the Ag NW surface was oxidized to AgO as the acidic electrolyte was adsorbed and penetrated into the PPy layer. In particular, after the cycle test, new peaks (202) and (132), which were not detected in Ag NW, were detected, and it was confirmed that these were caused by AgO generated via the oxidation of Ag.

## 4. Conclusions

Core–shell rod-shaped Ag NW@PPy nanomaterials were successfully synthesized using a simple suspension polymerization technique. Pseudomaterial layers (PPy) with controlled thickness and shell sequence were coated uniformly throughout the Ag NWs. The PPy layer on the Ag NW surface was effectively controlled by reaction conditions. The synergistic effect of each component (PPy and Ag NWs) in addition to the porous network structure composed of 1D conductive Ag NW@PPy provided excellent electrochemical performance. The 3D Ag NW@PPy network can significantly reduce the internal resistance of the electrode and maintain structural stability. As a result, we obtained high specific capacitances of 625 and 165 F/g at a scan rate of 1 mV/s for the Ag NW@10 nm PPy and Ag NW@60 nm PPy samples, respectively. We also evaluated the cycling performance of the 3D porous Ag NW@PPy composites over a long period (over 10,000 cycles) as an important key parameter for supercapacitor performance. We expect that our core–shell-structured Ag NW@PPy composites and their 3D porous structure network films can be applied as electrochemical materials for the design and manufacturing of supercapacitors and other energy storage devices.

## Data Availability

Data are contained within the article.
